# Normative Data for the Montreal Cognitive Assessment (MoCA) in Mexican Adults: A Regression-Based Approach

**DOI:** 10.3390/diagnostics15222920

**Published:** 2025-11-19

**Authors:** Lorena Parra-Rodríguez, Juan Silva-Pereyra, Sergio Sánchez-García, Carmen García-Peña, Juan Francisco Flores-Vázquez, Paloma Roa-Rojas

**Affiliations:** 1 Instituto Nacional de Geriatría, Ciudad de México 10200, Mexico; lparra@inger.gob.mx (L.P.-R.); mcgarcia@inger.gob.mx (C.G.-P.); jfflores@inger.gob.mx (J.F.F.-V.); 2 Facultad de Estudios Superiores Iztacala, Universidad Nacional Autónoma de México, Ciudad de México 04510, Mexico; juan.silva.pereyra@iztacala.unam.mx; 3 Unidad de Investigación Epidemiológica y en Servicios de Salud, Área Envejecimiento, Instituto Nacional de Seguro Social, Ciudad de México 06600, Mexico; sergio.sanchezga@imss.gob.mx

**Keywords:** Montreal Cognitive Assessment (MoCA), cognitive screening, normative data, age and education effects, Mexican population, regression-based norms

## Abstract

**Background/Objectives:** The Montreal Cognitive Assessment (MoCA) is a widely used cognitive screening tool designed to detect cognitive impairment. However, evidence indicates that the original cut-off score of 26 and the one-point correction for low education may not be appropriate across diverse populations. In Latin America, and particularly in Mexico, existing validation studies are scarce and limited by small sample sizes. The objective of this study was to examine the effects of age and education on MoCA performance in Mexican adults and to develop regression-based normative data for more accurate interpretation. **Methods:** MoCA performance of 2546 cognitively healthy participants aged 18–99 years from two public health institutions in Mexico City was analyzed. Inclusion criteria required preserved cognition, functionality, independence, and absence of conditions directly affecting brain health. The Spanish version 8.1 of the MoCA was administered. Age-adjusted normative values were obtained. Then, regression analyses were applied to generate age- and education-adjusted norms. **Results:** MoCA total scores correlated negatively with age and positively with education, while sex showed no significant effect. Regression-based norms revealed that identical raw total scores have different normative interpretations depending on age and education. **Conclusions:** This study provides the first regression-based MoCA norms for Mexican adults, demonstrating that both age and education exert a substantial influence on test performance. These norms enable a more precise, culturally sensitive interpretation than fixed cut-off scores and reduce the risk of misclassification in clinical and research settings.

## 1. Introduction

The Montreal Cognitive Assessment (MoCA) [1] is a cognitive screening test widely used to detect cognitive impairment associated with multiple neurological conditions [2,3,4,5,6]. It assesses executive function, memory, orientation, visuospatial skills, abstraction, and language via 10 subtests and yields a global cognition score ranging from 0 to 30, this being the maximum score. The MoCA has been validated in several countries and translated into multiple languages [7,8]. In the original study [1], the MoCA was proposed to detect age-related cognitive impairment. The authors examined 277 older adults assigned to three groups: mild Alzheimer-type dementia, mild cognitive impairment (MCI), and cognitively normal controls. Receiver-operating characteristics supported a 26-point cut-off score. An education adjustment was also proposed, by adding one point to the total score for individuals with fewer than 12 years of formal education. However, substantial evidence indicates that the 26-point cut-off score may be too high for multiple neurological conditions such as MCI [9,10], stroke [11] or traumatic brain injury [12] and non-neurological conditions that affect cognition [13,14], and this education adjustment is insufficient [15,16].

The cut-off score being too high has also been found in several normative studies with cognitively healthy participants where the majority’s performance falls below this cut-off score [17,18,19,20]. Likewise, the original one-point education adjustment appears insufficient for populations with varied levels of education such as the Mexican population [21], since evidence suggests up to four points of adjustment [22]. Also, other studies suggest different cut-offs for each education level [23], even for highly educated subjects [24]. Taken together, these findings suggest a high likelihood of misclassification and underline the need for more precise measures to improve the interpretation of results.

Findings from Latin America resemble previous evidence showing that the original cut-off tends to be high (for MCI and other disorders) [25,26,27,28]. In Mexico, there is one MoCA study that has been published [29]. Its aim was to establish the validity of the MoCA for identifying MCI and dementia in an elderly Mexican population, using a sample of 168 participants classified into three groups: cognitively healthy, MCI, and dementia. The authors concluded that the MoCA is a reliable and valid instrument for screening MCI and dementia, and that the original cut-off score of 26 can be applied to Mexican older adults. However, this was not a normative study, and therefore it is limited by its small sample size and the absence of age- or education-specific adjustments.

Overall, current evidence shows a wide range of cut-off scores and education adjustments. This variability may reflect methodological limitations across studies [30] and the strong influence of cultural and sociodemographic factors—particularly age, sex and education—on performance [16,31,32]. The Mexican context underscores this influence considering the heterogeneous educational attainment of the population, highlighting the need to examine these factors and to propose context-specific adjustments in order to prevent misclassification errors and false positives. Accordingly, the aim of the present study was to analyze the effects of age, sex and education on MoCA performance and then, having ruled out the sex variable after this analysis, develop regression-based norms for cognitively healthy adults. We selected this approach because it allows for the introduction of important sociodemographic variables through multiple regression [33], and a more precise and nuanced interpretation of performance than traditional methods [34,35,36].

## 2. Materials and Methods

### 2.1. Participants

This is a retrospective analysis of data belonging to two previous studies from two public health institutions in Mexico City: the Mexican Social Security Institute (IMSS) and the National Institute of Geriatrics (INGER). From IMSS, data from 1252 participants from the Cohort of Obesity, Sarcopenia, and Frailty in Mexican Older Adults (COSFOMA) [37] were included. In summary, COSFOMA is a population-based prospective cohort initiated in 2014, comprising adults aged 60 years and older who were beneficiaries of IMSS. The main goal of this study is to gather annual clinical and sociodemographic data through interviews conducted by trained healthcare personnel. From INGER, data from 1517 participants aged 18 and older from a previous normative study were included. The main goal of this study was to obtain regression-based norms of common neuropsychological instruments. All data was collected between 2017 and 2021, yielding a total convenience sample of 2769 participants. A joint database was created with the following variables:

• For cognition, cognitive performance was assessed using the Trail Making Test (TMT) Parts A and B and verbal fluency tasks [38,39]. The TMT evaluates processing speed, visual attention, and cognitive flexibility, with performance expressed as total response time in seconds for each form (A and B); shorter times indicate better cognitive performance, with typical completion times ranging approximately from 20–80 s for Part A and 40–180 s for Part B, depending on age and education. Verbal fluency was measured through both semantic fluency (naming animals) and phonemic fluency (words beginning with the letter “P”) tasks, which assess lexical access and executive control. Scores correspond to the total number of correct words generated in 60 s, with higher scores reflecting better verbal fluency.
• For functional status, the Lawton–Brody Instrumental Activities of Daily Living (IADL) Scale and the Barthel Activities of Daily Living (ADL) Scale [40,41] were included. The Lawton–Brody IADL Scale evaluates independent functioning in complex daily tasks such as managing finances or medications, with scores ranging from 0 to 8, where higher values indicate greater independence. The Barthel ADL Scale assesses performance in basic self-care activities, such as feeding and mobility, with scores ranging from 0 to 100, where higher scores denote higher functional autonomy.
• For mood status, the Center for Epidemiologic Studies Depression Scale of seven items (CES-D 7) and the Yesavage Geriatric Depression Scale (GDS) [42,43] were used. CES-D 7 measures depressive symptoms in the general population, with scores ranging from 0 to 21, where higher scores reflect greater depressive symptomatology. The Yesavage GDS, applied to participants aged 60 years and older, ranges from 0 to 15, with higher scores indicating more severe depressive symptoms.
• For health status, a total of nine questions about the participants ever being diagnosed with any conditions that directly affect brain health were used. Conditions were: moderate or severe traumatic brain injury, brain tumor, epilepsy, stroke, schizophrenia or other primary psychotic disorders, clinically significant depression or other affective disorders, delirium, uncontrolled diabetes, or uncontrolled hypertension. Participants answered “yes” (=1) or “no” (=0). A dichotomous variable for each question or relevant medical condition was created, followed by a total score ranging from 0 to 9.
• For sociodemographic characteristics, age was used in years as a continuous variable, Gender was coded as a binary variable (female, male) and education was used also in years, as a continuous variable.

### 2.2. Procedure

Once the database was created, the following Inclusion criteria was applied for being considered “cognitively healthy”: (1) age ≥ 18 years; (2) no cognitive impairment based on neuropsychological test performance; which is cognitive total scores above 1.0 SD below mean age and education normative measures (verbal fluency and Trails A and B) [44,45], (3) totally preserved independence and functional status (Lawton score = 8, Barthel score = 100) [46,47]; (4) No depression (CESD-7 > o = 9, Yesavage > o = 11) [48,49], (5) no history of disease with a direct impact on brain health (total score = 0); (6) completion of all MoCA items; and (7) signed informed consent. After applying these criteria, the final sample comprised 2546 individuals. These inclusion criteria were designed to make certain the sample represented typical aging Mexican adults, meaning those who experience expected age-appropriate cognitive and functional changes [50].

### 2.3. MoCA Administration

The Spanish MoCA version 8.1 was administered to all subjects in the sample. This version assesses seven domains: orientation, abstraction, attention, memory, language, visuospatial function, and executive function. The test was administered and scored following the guidelines at www.mocatest.org. At INGER, assessments were conducted by qualified health personnel, meaning psychologists and physicians; at IMSS, assessments were conducted also by qualified health personnel and the MoCA test was administered by neuropsychologists.

### 2.4. Statistical Analysis

Statistical analyses were carried out using IBM SPSS Statistics Base 25.0. To study the effects of age, sex, and education, an initial analysis examined the relationship between the MoCA total score with demographic variables. Pearson coefficients of correlation (R) and determination (R^2^) were obtained [51]. Age and education were significantly correlated with the MOCA total score, but sex was not. Therefore, subsequent analyses used a multiple regression format to correct the scores for effects of age and education.

To obtain regression-based normative data, the following step-procedure was implemented [33,52]:

Age groups. For the creation of age groups, the overlapping interval strategy [53] was applied. This methodology defines overlapping age intervals around mid-points that increase in relatively small steps, five years in our case. This allows the maximization of the number of participants contributing to the normative distribution at each mid-point age interval.

MoCA Age-adjusted NOrmative Values (MANOV). For the construction of age-adjusted normative values [54,55,56], the frequency distributions of the raw scores of the MoCA were converted into the MoCA-age-adjusted normative values (MANOV) as follows: For each age group, a cumulative frequency distribution of the raw scores was generated. Raw scores were assigned their percentile considering their place within the distribution (percentile = (number of values below score) ÷ (total number of scores) 100). Subsequently, percentiles were scaled to a normalized distribution with mean of 10 and standard deviation (SD) 3 (MANOV = 3 invnormal(percentile ÷ 100) + 10, where invnormal is the inverse cumulative standard normal distribution, if normal(z) = p, then invnormal(p) = z). When percentiles were equal to zero, the age-adjusted normative value was equal to zero.

MoCA Education and Age-adjusted NOrmative Values (ME&ANOV). Linear regressions were implemented for the construction of education- and age-adjusted normative values [57]. The MANOV values were used as dependent variables, while years of education were used as an independent variable. For this linear regression model, we obtained the next equation:

MANOV = k + B(years of education) + ε,

where k is the constant of the model, B is the regression coefficient for years of education, and ε is the error of the model, then

ε = MANOV − k − B(years of education),

where ε follows a normal distribution with mean μ and SD σ. We rescaled this result to a normal distribution with mean = 10 and SD = 3 to obtain a distribution comparable to MANOV. We called this re-scaling the MoCA Education and Age normative value (ME&ANOV).

ME&ANOV = 10 + 3 (SSA − k − B(years of education) − μ) ÷ σ.

Although this rescaling choice is conventional rather than inherent to the data, it aligns with established neuropsychological norms [52] and facilitates clinical interpretation. Scores around 10 represent average performance, whereas deviations of ±3 or ±6 points correspond to one or two standard deviations below or above the mean, providing a simple and familiar framework for identifying atypical results.

**Figure 1:**
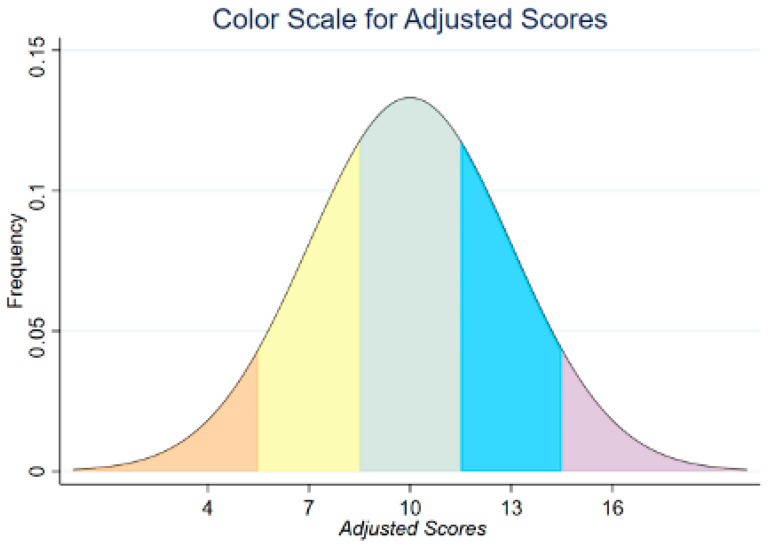
Color scale for adjusted scores. The curve represents a theoretical normal distribution (mean = 10, SD = 3). The figure illustrates how adjusted score ranges are color-coded and does not depict the empirical distribution of the study sample.

Finally, for making the tables, Microsoft Excel was used to compute the means for each age group of the MANOV and each education group of the ME&ANOV. For the interpretation of these tables, a color scale was applied to the normative values. This color scale is presented in Figure 1. The color scale goes as follows: orange equals scores around two standard deviations below the mean, yellow equals scores around one standard deviation below the mean, gray equals scores around the mean, blue equals scores around one standard deviation above the mean and purple equals scores around two standard deviations above the mean.

## 3. Results

### 3.1. Sociodemographic Characteristics

This study was approved by the Health Research Ethics Committee of IMSS (Registration Number: 2012-785-067). In the MoCA sample, 56.56% of participants were women. The mean age of the MoCA sample participants was 57.2 (SD = 17.2) years and average years of education was 12.4 (SD = 4.8) years. Sociodemographic characteristics of the whole sample are summarized in Table 1.

Through the overlapping interval strategy, thirteen mid-point age groups were obtained: 19–39, 24–44, 29–49, 34–54, 39–59, 44–64, 49–69, 54–74, 59–79, 64–84, 69–89, 74–94, and 79–99 years of age. Results and sample sizes for each age group are shown in Table 2. For example, norms for midpoint age 69 apply to ages 67–71 and are derived from all individuals between the ages of 59–79. These group conformations are presented in Table 2.

### 3.2. Total MoCA Scores, Age Education and Sex Correlations

The mean total MoCA score for the whole sample was 23.8 (SD = 4.6). Only 47.7% of subjects performed above the 26-point cut-off score. Age (R = −0.499, R^2^ = 0.249, *p* < 0.001) and years of education (R = 0.534, R^2^ = 0.285, *p* < 0.001) had moderate correlations with the MoCA total score. Sex had no significant association (R = −0.120, R^2^ = 0.014, *p* < 0.001). Because of the lack of significant R values between sex and MoCA scores, sex was not considered for regression analyses.

### 3.3. Age-Adjusted Normative Values

**Figure 2:**
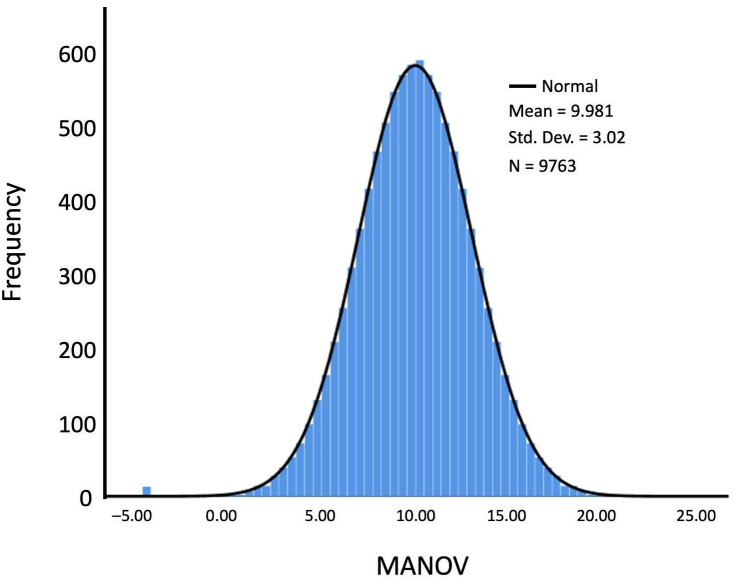
MANOV normalized distribution Histogram of the empirical MANOV values with an overlaid fitted normal density curve.

Age-adjusted normative values are presented in Table 3. In this table, each row corresponds to a raw score (1–30), while the columns represent age intervals in years (≤31, 32–36, 37–41, …, ≥87). The cell values provide standardized normative scores, expressed on a scale with a mean of 10 and a standard deviation of 3, which represent the expected performance for each combination of age and raw score. The MANOV scores followed a normalized distribution, as illustrated in Figure 2 (mean = 10; SD = 3; Kolmogorov–Smirnov test, *p* = 0.20).

To illustrate, consider the following examples: A 70-year-old person obtains a raw MoCA score of 22. Locating the corresponding row (raw score 22) and the column for the 67–71 age group yields a normative value of 13. This score lies above the reference mean (10) at approximately +1 standard deviation, indicating better-than-average performance for the age group, suggesting normal or even superior cognitive functioning. In contrast, a 40-year-old person with the same raw score (22) yields a normative value of 7, which corresponds to −1 standard deviation. This result falls below the expected for that age group, suggesting possible cognitive impairment and signaling the need for a more detailed clinical assessment. According to these results, the same raw score (22) has different implications depending on age: in an older adult (70 years), it reflects performance within or above expectations, whereas in a younger adult (40 years), it suggests below-average functioning and potential cognitive impairment.

### 3.4. Education- and Age-Adjusted Normative Values

**Figure 3:**
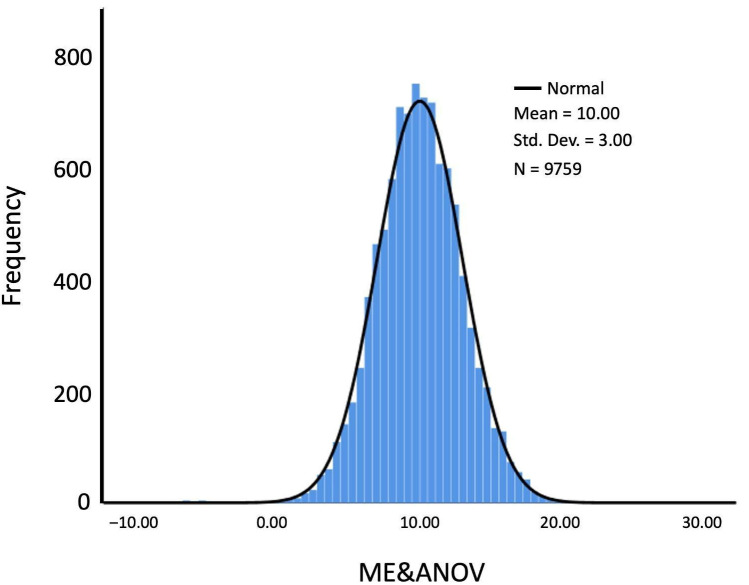
ME&ANOV normalized distribution. Histogram of the empirical ME&ANOV values with an overlaid fitted normal density curve.

Education- and age-adjusted normative values for MoCA are presented in Table 4, Table 5, Table 6 and Table 7. Years of education were categorized into four groups as follows: 0 years of education = no education, 1–6 years of education = basic education, 7–12 years of education = intermediate education, and 13 years of education or more = superior education or higher. For this transformation, the linear regression yielded a coefficient of correlation R of 0.378 (F = 19,757, df =1629.371, *p* < 0.001). Years of education (t = 40.365; *p* < 0.001; beta = 0.4) was a significant predictor and ε followed a normal distribution as shown in Figure 3 (Mean = −0.0029, SD = 2.7970). The equation to obtain the ME&ANOV was:

ME&ANOV = 10 + 3 (MOCA-ANOV − 7.260 − 0.228(years of education) + 0.0029) ÷ 2.79707.

Let us revisit the example of a 70-year-old person who obtains a total MoCA score of 22 and has 4 years of schooling (basic education). The corresponding normative score for this combination is 13, which is approximately +1 standard deviation above the mean. This indicates performance higher than expected for this demographic group.

Now consider a 40-year-old person with the same raw MoCA score (22) and the same educational level (basic). In this case, the normative score is 6, which is approximately −1 standard deviation. This result reflects below-expected performance, suggesting possible cognitive impairment. From this example, we can infer that adjusting by age and adjusting by both age and education produce nearly the same normative value. However, when the same example is considered with a 70-year-old person holding a university-level education, the normative score corresponding to a total of 22 is 8. In this case, after adjusting for age and education, performance falls below expectations, suggesting probable cognitive impairment. Similarly, a 40-year-old adult with university-level education who scores 22 obtains a normative value of 3, which also indicates markedly reduced performance. In these cases, when adjusting for age and education, the same total raw score of 22 reflects substantially lower cognitive performance in comparison to the age-adjusted normative values.

For the interpretation of performance, there is inherent variability in how an individual’s score is interpreted relative to the normative mean. However, a commonly used framework classifies scores within one standard deviation of the mean as broadly average. Scores between one and two standard deviations below the mean are generally considered mildly impaired, while those falling two or more standard deviations below the mean are typically interpreted as indicating at least moderate impairment [58,59]. Applying this framework to the previous examples, a MoCA score of 22 of a 40-year-old person with elementary educational level could be interpreted as mildly impaired and a MoCA score of 22 of a 70-year-old person with university educational level could be interpreted also as mildly impaired.

## 4. Discussion

The MoCA is a screening test commonly used to detect cognitive impairment in individuals with various neurological disorders. Evidence shows there are a wide variety of cut-off scores and education adjustments, mainly because of age and education specific effects, signaling the need for context-specific calibration [30]. Hence, our main goal was to examine the effects of age and education on MoCA performance in cognitively healthy Mexican adults and then obtain regression-based norms. In sum, our findings suggest interpreting the MoCA total score via a single cut-off could lead to misclassification errors. Regression-based, age- and education-adjusted norms enable a far more precise and context-sensitive interpretation, reducing the risk of false positives, especially in low-education populations such as adults in Mexico and across Latin America.

As expected, correlation analyses showed a negative association between age and total score: as age increases, scores decrease, with the oldest participants showing the lowest performance. This aligns with prior work [60], including studies with very old adults [61]. Evidence points to an age-related decline in global cognition, consistent with the exponential rise in MCI incidence across age [62]. Numerous studies highlight the MoCA’s utility for detecting MCI [63], and some evidence suggests it outperforms other instruments for this purpose [26,64]. In contrast, education correlated positively with performance: higher educational attainment was associated with higher scores, a finding previously reported even among highly educated older adults [24].

In the original validation [1], a 26-point cut-off score was proposed. In our data, this threshold was high: only 47.7% of the total sample scored above it and among those over 60 years, only 30.2% exceeded this threshold. These results are consistent with prior reports and suggest that a single cut-off score is inadequate [11]. Age-adjusted normative values further show that the same MoCA total score may be acceptable in older adults yet indicate low performance in younger adults. From a clinical standpoint, these discrepancies highlight the risk of both over- and under-identifying cognitive impairment when age-specific norms are not applied. For example, a total score of 22 corresponds to normal-to-high performance in a 70-year-old (normative score = 13), but to a moderate deficit in a 40-year-old (normative score = 7) reflecting expected age-related cognitive changes and heterogeneous aging trajectories even on a brief tool like the MoCA.

The original MoCA study also recommended adding one point for individuals with <12 years of education [1]. Applying that rule here, only 31.6% of participants surpassed the 26-point cut-off score, highlighting its inadequacy in populations with broad educational ranges such as in Mexico. With the regression-based approach, young highly educated adults showed the highest normative values meaning they would require larger education adjustments, whereas highly educated older adults had lower normative values meaning they would require fewer points of adjustment. For example, a total score of 22/30 in a university-educated 70-year-old adult, indicates a moderate deficit (normative score = 8), whereas in a university-educated 40-year-old adult, it indicates a severe deficit (normative score = 3). These results align with studies recommending larger education adjustments for participants with lower schooling [22].

Strengths of this study include the large sample, arguably the largest MoCA normalization study to date in Latin America; and the broad age range, which allowed examination of sociodemographic effects across generations. This study is also the first, to our knowledge, to offer regression-based MoCA norms for Mexico, a robust approach that goes beyond stratification. Furthermore, the tables support interpretation adjusted by age alone or by age plus education, providing a rigorous framework for detecting cognitive impairment. Color-coded outputs also help users to quickly situate an individual’s performance relative to the reference group. Limitations include this is a convenience sample; therefore, although the inclusion criteria and the large sample size reflect typical characteristics of the Mexican population, it cannot be considered statistically representative. Accordingly, the generalization of these findings to the broader population should be made with caution. Also, reliance primarily on self-reported health histories and the absence of neuroimaging and other biomarkers to appraise possible underlying brain disease. Finally, MoCA is a screening instrument; results should be interpreted cautiously, particularly in clinical settings.

Clinicians can integrate these norms into practice by interpreting MoCA scores relative to expected performance for a given group, rather than applying a single universal cut-off score. Doing so helps distinguish normal age-related cognitive changes from clinically meaningful cognitive decline and the influence of a wide range of educational levels on cognitive performance, reducing the risk of false positives in older adults and false negatives in younger adults. Incorporating normative values into electronic health records, decision-support tools, and routine cognitive screening workflows may further facilitate their use in busy primary care settings.

Future studies should validate these norms to ensure their accuracy, particularly in low- and middle-income countries like Mexico, where cognitive impairment trajectories may differ. Longitudinal research is also needed to determine how normative scores predict clinical outcomes such as progression from MCI to dementia. Additionally, examining how specific neurological conditions interact with these norms may refine diagnostic accuracy and enhance more personalized cognitive assessments.

## 5. Conclusions

In conclusion, age and education affect MoCA performance in cognitively healthy Mexican adults. Adjusting scores for these factors yields values that differ from previous studies, highlighting the importance of using population-appropriate norms for accurate interpretation. Our findings are consistent with the prior literature and support refining MoCA scoring by incorporating demographic influences on cognitive performance. Given the non-clinical nature of this sample, future work should conduct full sensitivity and specificity analyses to determine how well these proposed norms translate to clinical populations.

## Figures and Tables

**Table 1:** Sociodemographic characteristics.

Total Sample (*n*)	2546
Sex (%)	
Male	43.44
Female	56.56
Age (%)	
18–34 years	15.67
35–54 years	17.05
55–64 years	32.60
65–74 years	21.01
75–99 years	13.67
Education (%)	
Basic (6 or fewer years)	22.02
Intermediate (7 until 12 years)	32.10
Superior (13 to 15 years)	32.68
Above superior (more than 15 years)	13.20

**Table 2:** MoCA mid-point age groups (*n* = 2546).

Groups	Mid-Point Age	Age Range for Mid-Points	Age Range for Norms	Sample Size MOCA
1	29	≤31	19–39	452
2	34	32–36	24–44	368
3	39	37–41	29–49	380
4	44	42–46	34–54	369
5	49	47–51	39–59	370
6	54	52–56	44–64	1099
7	59	57–61	49–69	1462
8	64	62–66	54–74	1473
9	69	67–71	59–79	1595
10	74	72–76	64–84	1073
11	79	77–81	69–89	545
12	84	82–86	74–94	384
13	89	≥87	79–99	193

**Table 3:** Age-adjusted normative values (MANOV).

MOCA	Age (Years)												
Score	≤31	32–36	37–41	42–46	47–51	52–56	57–61	62–66	67–71	72–76	77–81	82–86	≥87
1			** **	** **	** **	** **	** **	** **	0	** **	0	0	** **
2									0		1	2	
3													
4									1		2	2	0
5									1	2	3	3	2
6							0	0	2	2	3	3	3
7						0	1	1	2	3	3	4	4
8							2	2	3	3	4	4	5
9						2	2	3	3	4	4	5	5
10						3	3	3	4	4	5	5	6
11						3	3	3	4	4	5	6	6
12						3	3	4	4	5	6	6	6
13						4	4	4	5	5	6	7	7
14						4	4	5	5	6	7	7	8
15						5	5	5	5	6	7	8	8
16						5	5	5	6	7	8	8	8
17				0	0	5	6	6	6	7	8	9	9
18				2	2	6	6	7	7	8	8	9	9
19	0	1	1	3	3	7	7	7	8	8	9	9	10
20			3	4	4	7	8	8	8	9	9	10	10
21	2	3	4	5	5	8	8	8	9	9	10	10	11
22	3	4	5	5	6	8	9	9	9	10	10	11	11
23	4	5	5	6	6	9	9	9	10	10	11	11	12
24	5	5	6	6	7	9	10	10	10	11	11	12	12
25	6	6	7	7	8	10	11	11	11	12	12	12	13
26	8	8	8	9	9	11	11	12	12	13	13	13	14
27	9	9	10	11	11	12	13	13	13	14	14	15	15
28	11	11	12	12	12	14	14	14	15	15	15	16	16
29	13	13	13	14	14	15	16	16	16	16	17	17	17
30	16	16	16	16	17	18	18	18	18	18	19	18	18

**Table 4:** Education- and age-adjusted normative values—No education (0 years).

MOCA	Age (Years)												
Score	≤31	32–36	37–41	42–46	47–51	52–56	57–61	62–66	67–71	72–76	77–81	82–86	≥87
1									2		2	2	
2									3		4	4	
3													
4									3		4	5	2
5									4	4	5	5	5
6							2	2	4	5	5	6	6
7						3	3	3	5	5	6	6	7
8							4	4	5	6	6	7	7
9						4	5	5	6	6	7	7	8
10						5	5	5	6	7	7	8	8
11						5	5	6	6	7	8	8	8
12						6	6	6	7	7	8	9	9
13						6	6	7	7	8	9	9	10
14						7	7	7	8	8	9	10	10
15						7	7	8	8	9	10	10	11
16						8	8	8	9	9	10	11	11
17				2	2	8	8	9	9	10	11	11	12
18				4	4	9	9	9	10	10	11	12	12
19	2	3	3	5	5	9	10	10	10	11	12	12	13
20			5	6	6	10	10	10	11	11	12	13	13
21	4	6	6	7	7	11	11	11	11	12	13	13	14
22	6	6	7	8	8	11	11	12	12	12	13	14	14
23	7	7	8	9	9	12	12	12	13	13	14	14	15
24	8	8	8	9	10	12	13	13	13	14	15	15	15
25	9	9	9	10	10	13	13	14	14	15	15	16	16
26	10	10	11	12	12	14	15	15	15	16	16	17	17
27	12	12	13	14	14	16	16	16	17	17	18	18	18
28	14	14	15	15	15	17	17	18	18	18	19	19	19
29	16	16	16	17	17	19	19	19	20	20	21	21	20
30	20	19	19	20	20	21	22	22	22	22	22	22	21

**Table 5:** Education- and age-adjusted normative values—Basic education (1 to 6 years).

MOCA	Age (Years)												
Score	≤31	32–36	37–41	42–46	47–51	52–56	57–61	62–66	67–71	72–76	77–81	82–86	≥87
1									1		1	1	
2									2		3	3	
3													
4									2		3	4	1
5									3	3	4	4	4
6							1	1	3	4	5	5	5
7						2	2	2	4	4	5	6	6
8							3	3	4	5	6	6	6
9						3	4	4	5	5	6	7	7
10						4	4	5	5	6	6	7	7
11						4	5	5	5	6	7	7	8
12						5	5	5	6	7	7	8	8
13						5	5	6	6	7	8	9	9
14						6	6	6	7	8	8	9	10
15						6	6	7	7	8	9	10	10
16						7	7	7	8	9	9	10	10
17				1	1	7	8	8	8	9	10	11	11
18				3	3	8	8	8	9	9	10	11	11
19	1	3	3	4	4	9	9	9	9	10	11	12	12
20			4	5	6	9	9	10	10	10	11	12	12
21	4	5	5	6	7	10	10	10	11	11	12	13	13
22	5	6	6	7	7	10	11	11	11	12	13	13	14
23	6	6	7	8	8	11	11	11	12	12	13	14	14
24	7	7	8	8	9	11	12	12	13	13	14	14	14
25	8	8	8	9	9	12	13	13	13	14	14	15	15
26	9	10	10	11	11	13	14	14	14	15	15	16	16
27	11	11	12	13	13	15	15	15	16	16	17	17	17
28	13	13	14	14	15	16	16	17	17	17	18	18	18
29	15	15	15	16	16	18	18	18	19	19	20	20	19
30	19	19	19	19	19	20	21	21	21	21	21	21	20

**Table 6:** Education- and age-adjusted normative values—Intermediate education (7 to 12 years).

MOCA	Age (Years)												
Score	≤31	32–36	37–41	42–46	47–51	52–56	57–61	62–66	67–71	72–76	77–81	82–86	≥87
1									0		0	0	
2									0		1	2	
3													
4									1		2	2	0
5									1	2	3	3	2
6							0	0	2	2	3	4	3
7						0	1	1	2	3	4	4	4
8							2	2	3	3	4	5	5
9						2	2	3	3	4	5	5	5
10						3	3	3	4	4	5	6	6
11						3	3	3	4	5	5	6	6
12						3	3	4	4	5	6	7	7
13						4	4	4	5	6	7	7	8
14						4	5	5	5	6	7	8	8
15						5	5	5	6	7	7	8	8
16						5	6	6	6	7	8	9	9
17				0	0	6	6	6	7	8	9	9	10
18				2	2	6	7	7	7	8	9	10	10
19	0	1	1	3	3	7	7	8	8	8	9	10	10
20			3	4	4	8	8	8	9	9	10	11	11
21	2	3	4	5	5	8	9	9	9	10	10	11	12
22	3	4	5	6	6	9	9	9	10	10	11	12	12
23	4	5	6	6	7	9	10	10	10	11	12	12	13
24	5	6	6	7	7	10	10	11	11	11	12	13	13
25	6	7	7	8	8	11	11	11	12	12	13	13	14
26	8	8	9	9	10	12	12	12	13	13	14	14	15
27	10	10	11	11	11	13	14	14	14	15	15	16	16
28	12	12	12	13	13	15	15	15	16	16	16	17	17
29	14	13	14	15	15	16	17	17	17	17	18	18	18
30	17	17	17	18	18	19	19	19	20	20	20	20	19

**Table 7:** Education- and age-adjusted normative values—Superior education (>12 years).

MOCA	Age (Years)												
Score	≤31	32–36	37–41	42–46	47–51	52–56	57–61	62–66	67–71	72–76	77–81	82–86	≥87
1									0		0	0	
2									0		0	0	
3													
4									0		1	1	0
5									0	0	1	2	1
6							0	0	0	1	2	2	2
7						0	−1	−1	1	1	2	3	3
8							0	1	1	2	3	3	3
9						0	1	1	2	3	3	4	4
10						1	1	2	2	3	4	4	4
11						1	2	2	3	3	4	4	5
12						2	2	2	3	4	4	5	5
13						2	2	3	3	4	5	6	6
14						3	3	3	4	5	6	6	7
15						3	4	4	4	5	6	7	7
16						4	4	4	5	6	6	7	7
17				0	0	4	5	5	5	6	7	8	8
18				0	0	5	5	5	6	7	7	8	8
19	0	0	0	2	1	6	6	6	7	7	8	9	9
20			2	3	3	6	7	7	7	8	8	9	10
21	1	2	2	3	4	7	7	7	8	8	9	10	10
22	2	3	3	4	5	7	8	8	8	9	10	10	11
23	3	4	4	5	5	8	8	8	9	9	10	11	11
24	4	4	5	5	6	8	9	9	10	10	11	11	12
25	5	5	5	6	7	9	10	10	10	11	11	12	12
26	6	7	7	8	8	10	11	11	11	12	12	13	13
27	8	8	9	10	10	12	12	12	13	13	14	14	14
28	10	10	11	12	12	13	14	14	14	14	15	15	15
29	12	12	12	13	13	15	15	15	16	16	17	17	16
30	16	16	16	16	16	17	18	18	18	18	19	18	17

## Data Availability

The datasets presented in this article are not readily available because of ethics restrictions. Requests to access the datasets should be directed to projas@inger.gob.mx.
